# The feasibility and added value of mapping music during awake craniotomy: A systematic review

**DOI:** 10.1111/ejn.15559

**Published:** 2021-12-28

**Authors:** Pablo R. Kappen, Tobia Beshay, Arnaud J. P. E. Vincent, Djaina Satoer, Clemens M. F. Dirven, Johannes Jeekel, Markus Klimek

**Affiliations:** ^1^ Neurosurgery Erasmus MC Rotterdam Netherlands; ^2^ Surgery Erasmus MC Rotterdam Netherlands; ^3^ Neuroscience Erasmus MC Rotterdam Netherlands; ^4^ Anesthesiology Erasmus MC Rotterdam Netherlands

**Keywords:** awake craniotomy, brain mapping, music performance

## Abstract

The value of mapping musical function during awake craniotomy is unclear. Hence, this systematic review was conducted to examine the feasibility and added value of music mapping in patients undergoing awake craniotomy. An extensive search, on 26 March 2021, in four electronic databases (Medline, Embase, Web of Science and Cochrane CENTRAL register of trials), using synonyms of the words “Awake Craniotomy” and “Music Performance,” was conducted. Patients performing music while undergoing awake craniotomy were independently included by two reviewers. This search resulted in 10 studies and 14 patients. Intra‐operative mapping of musical function was successful in 13 out of 14 patients. Isolated music disruption, defined as disruption during music tasks with intact language/speech and/or motor functions, was identified in two patients in the right superior temporal gyrus, one patient in the right and one patient in the left middle frontal gyrus and one patient in the left medial temporal gyrus. Pre‐operative functional MRI confirmed these localizations in three patients. Assessment of post‐operative musical function, only conducted in seven patients by means of standardized (57%) and non‐standardized (43%) tools, report no loss of musical function. With these results, we conclude that mapping music is feasible during awake craniotomy. Moreover, we identified certain brain regions relevant for music production and detected no decline during follow‐up, suggesting an added value of mapping musicality during awake craniotomy. A systematic approach to map musicality should be implemented, to improve current knowledge on the added value of mapping musicality during awake craniotomy.

## Graphical abstract

In this systematic review, we found that intra‐operative mapping of music was successful in 13 out of 14 patients. We identified regions responsible for isolated music function, music combined with motor function and music combined with language/speech function. No decline of post‐operative musical function, assessed in seven (50%) patients, was reported.

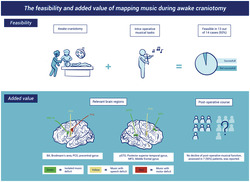

**Abbreviations**
BAT: Beat Alignment Test
fMRI: functional MRI
IQR: interquartile range
MBEA: Montreal Battery of Evaluation of Amusia
MEPS/SSEPS: motor and somatosensory‐evoked potentials
MFG: middle frontal gyrus
PRISMA: Reporting Items for Systematic Reviews and Meta‐analysis
SD: standard deviation
SMA: supplementary motor area
SRT: Seashore Rhythm Test
STG: superior temporal gyrus

## 1 INTRODUCTION

Neurosurgical procedures include surgery near brain regions responsible for patients' motor, speech or language function (so‐called eloquent brain regions) (Fugate, 2015). Awake craniotomy is applied when operating near these eloquent structures to safely remove tumour or epileptogenic zones, while monitoring patients' speech, language or motor functions (Penfield, 1937).

Musicians occasionally undergo awake craniotomy, during which their musical ability is at stake (Gasenzer et al., 2017a). Preservation of musical function may be of major importance for these patients as music can act as a main source of income (Bittman et al., 2005). Furthermore, loss of musical ability may have a severe impact on their quality of life, since music can serve as an outlet for emotions and contributes to the reduction of stress and anxiety (Bittman et al., 2005; de Witte et al., 2020).

Musical function, independent of speech/language or motor function, is usually not monitored during awake craniotomy. Relevant brain regions for music production include the premotor, prefrontal and supplementary motor cortices, along with the cerebellum, basal ganglia and the auditory superior temporal gyrus (STG) as these regions enable the auditory‐motor interactions required for music production (Gaser & Schlaug, 2003; Leonard et al., 2019; Zatorre et al., 2007). Moreover, the right hemisphere, which is mainly responsible for melodic identification, and the left auditory cortex, essential for the discrimination of speech/language, are in constant dialogue with one another through the corpus callosum (Hyde et al., 2009; Lee et al., 2003; Ozturk et al., 2002; Schlaug et al., 1995; Schneider et al., 2005; Zatorre et al., 2007).

Mapping music tasks, additional to speech/language and motor function, during awake craniotomy might be valuable, as focal damage within the right STG has shown to disrupt musical processing, without interfering with speech/language or motor functions (Gaser & Schlaug, 2003). Furthermore, post‐operative amusia (i.e., the inability to produce music) has already been described after right‐sided resection of a glioma (Russell & Golfinos, 2003). Hence, several case studies and video reports on social media, summarized in a previous narrative review, report patients performing music during awake craniotomy (Bass et al., 2020; Leonardi et al., 2018; Roux et al., 2009; Scerrati, Labanti, et al., 2020).

No systematic review of literature has been published addressing the feasibility and added value of intra‐operative music tasks during awake craniotomy. A clear and specific overview of the intra‐operative music mapping methods, the relevant brain regions and the peri‐operative course of musicality could serve as a guidance in clinic and for future studies.

## 2 METHODS

This systematic review follows the guideline from Reporting Items for Systematic Reviews and Meta‐analysis (PRISMA) and is registered in the PROSPERO database (CRD42021261017) (Moher et al., 2009).

### 2.1 Ethics

Informed consent or approval from the local institutional review board was not required for this systematic review, as no animals or patients were involved in the process.

### 2.2 Search strategy and eligibility criteria

The literature search was conducted with assistance of a dedicated biomedical information specialist. The electronic databases of Medline, Embase, Web of Science and Cochrane CENTRAL register of trials were searched from the date of inception until 26 March 2021, using terms and synonyms of the words “Awake Craniotomy” and “Music Performance” (Appendix A) (Bramer et al., 2018). Cross‐reference was applied in the included studies to search for additional eligible papers.

Prospective, retrospective cohort studies and case series/reports including patients performing music (i.e., humming/singing or any instrument) while undergoing awake craniotomy were included. Articles were excluded when full text was not available.

### 2.3 Source selection

Two independent reviewers (P.K. and T.B) screened all studies on title, abstract and full text when eligible. Discrepancies were discussed with the senior author (M.K.) until consensus was reached. Authors were not contacted to acquire additional information, since the aim of this review was to present an unmodified overview of the current literature.

### 2.4 Data extraction

Demographic patient data (i.e., age, sex and handedness), musicality (i.e., professional/amateur), type of musician (singer/instrumentalist), disease information (i.e., location/type/hemispheric side), course of musicality in comparison with speech/language and motor function (i.e., standardized/non‐standardized pre‐ and post‐operative tests), specifications of the intra‐operative mapping procedure (i.e., type of music/language/motor tasks, stimulation settings and mapped brain regions) and surgical details (i.e., anaesthesia technique, surgical course, occurrence of complications) were independently extracted by the same two reviewers (P.K. and T.B). Full text was again accessed when differences in data between the two independent reviewers were identified.

Level of musicality was not further specified, but rather adopted as stated by the authors of the included studies, using terms as “professional” and “non‐professional (e.g., hobbyist/amateur/casual player).

Successful intra‐operative mapping of music was defined as performance of intra‐operative music tasks, while using direct electrical stimulation for mapping purposes, without onset of task‐related surgical complications.

Intra‐operative findings during music mapping were categorized based on the localization of brain mapping on (sub)lobar level and severity of the disruption classified in major (e.g., complete music arrest) and minor (e.g., changes in pitch/rhythm/melodic contour) errors. Intra‐operative disruption during music tasks *without* reporting motor and/or speech/language deficits was classified as “isolated.” Intra‐operative disruption during music tasks *with* deficits in the same region during speech/language tasks and/or observed motor deficits was classified as “combined.”

Assessment of pre‐ and post‐operative musical function was defined as “standardized” in case of an objective scoring system, which has been published in a scientific journal (e.g., just mentioning playing the guitar would qualify for “non‐standardized”).

### 2.5 Data analysis and synthesis

Data were reported with mean ± standard deviation (SD) in normal distributed data (assessed with the Shapiro–Wilk test) or median and interquartile range (IQR) in non‐normal distributed data (Shapiro & Wilk, 1965).

BrainVoyager EDU (Brain Innovation, Maastricht, The Netherlands) was used for the quantitative visualization of the brain regions relevant for music mapping. Only cases which sufficiently specified these regions (i.e., with illustration) were included in this figure (Goebel et al., 2006).

## 3 RESULTS

### 3.1 Systematic search

The literature search generated 660 studies after removal of duplications (Appendix B). We excluded 642 studies after title and abstract screening, resulting in 18 studies to be assessed for full‐text. We excluded nine studies after full‐text screening: six studies due to a lack of intra‐operative music performance (Gasenzer et al., 2017b; Gayoso García et al., 2015; Riva et al., 2016; Roux et al., 2007; Schulz et al., 2005; Suarez et al., 2010), one conference abstract (Gripp et al., 2017), one case sang unexpectedly after stimulation but not for mapping purposes (Herbet et al., 2015) and one case (Breshears et al., 2019) due to overlap with another included study (Leonard et al., 2019). Cross‐referencing led to one additional study (Zhang et al., 2013) resulting in 10 studies (n_s_) and 14 patients (n_c_) included for the final analysis.

### 3.2 Study and patient characteristics

Mean/SD age of the 14 included patients was 38.57/16.05, of which nine male (64.3%) and 12 right‐handed patients (85%, Table 1). Eight patients were singers (57%) (Bass et al., 2020; Katlowitz et al., 2017; Roux et al., 2009; Zhang et al., 2013), while others played either a string (*n* = 4, 29%) (Bass et al., 2020; Dziedzic et al., 2020; Hegde et al., 2016; Leonard et al., 2019; Piai et al., 2018) or a wind instrument (*n* = 2, 14.3%). Six out of 14 patients were professional musicians (43%) (Garcea et al., 2017; Hegde et al., 2016; Leonard et al., 2019; Piai et al., 2018; Scerrati, Mongardi, et al., 2020).

**TABLE 1:** Demographic patient data

Authors	Case code	Gender	Age	Handness	Disease	Side	Disease location	Professional^a^	Instrument
Bass et al., 2020	B	F	19	R‐H	Glioneural	Right	Temporal	No	Singer
Dziedzic et al., 2020	D	F	19	R‐H	Cavernous malformation	Left	Temporal	No	Pianist
Garcea et al., 2017	G	M	26	R‐H	Tumour	Right	STG/MTG	Yes	Saxophonist
Hegde et al., 2016	H	M	16	R‐H	Epilepsy	Right	MTG	Yes	Violinist and singer
Katlowitz et al., 2017	K	M	41	R‐H	Epilepsy	Right	Temporal	Yes	Singer
Leonard et al., 2019	L	M	25	R‐H	Astrocytoma	Left	Insula	Yes	Guitarist
Piai et al., 2018	P	NR^b^	35–40	L‐H	Oligodendroglioma	Left	SMA	Yes	Violinist
Roux et al., 2009 (1)	R1	M	37	R‐H	Astrocytoma	Left	Frontal	No	Singer
Roux et al., 2009 (2)	R2	M	37–68^c^	R‐H	Tumour	Left	Frontal	No	Singer
Roux et al., 2009 (3)	R3	M	70	R‐H	Metastasis	Right	Frontal	No	Singer
Roux et al., 2009 (4)	R4	M	37–68^c^	R‐H	Tumour	Right	Frontal	No	Singer
Roux et al., 2009 (5)	R5	M	37–68^c^	L‐H	Tumour	Right	SMA	No	Singer
Scerrati, Mongardi, et al., 2020	S	F	52	R‐H	GBM	Right	Parietal Rolandic	Yes	Clarinetist
Zhang et al., 2013	Z	F	19	R‐H	GBM	Left	MFG	No	Singer

Abbreviations: F, female; GBM; glioblastoma multiforme; L‐H, left‐handed; M, male; MTG, middle temporal gyrus; R‐H, right‐handed; SMA, supplementary motor area; STG, superior temporal gyrus.

^a^ Level of musicality (i.e., professional vs. amateur) was adopted from the included studies.

^b^ Not reported.

^c^ In Roux et al., not all cases were separately described; hence, age could not always be determined.

Eleven patients underwent awake craniotomy for tumour resection (79%) (Bass et al., 2020; Garcea et al., 2017; Leonard et al., 2019; Piai et al., 2018; Roux et al., 2009; Scerrati, Mongardi, et al., 2020; Zhang et al., 2013), two for epilepsy surgery (14%) (Hegde et al., 2016; Katlowitz et al., 2017) and one because of a cerebral cavernous malformation (7%) (Dziedzic et al., 2020). Disease localization (right hemisphere, *n* = 8) was present in the temporal (*n* = 5, 36%) (Bass et al., 2020; Dziedzic et al., 2020; Garcea et al., 2017; Hegde et al., 2016; Katlowitz et al., 2017), frontal (*n* = 7, 50%) (Piai et al., 2018; Roux et al., 2009; Zhang et al., 2013) and parietal lobes (*n* = 1, 7%) (Scerrati, Mongardi, et al., 2020) and insula (*n* = 1, 7%) (Leonard et al., 2019).

Disease‐related seizures were reported in nine cases, but further no neurological deficits were described at baseline (Bass et al., 2020; Dziedzic et al., 2020; Garcea et al., 2017; Hegde et al., 2016; Katlowitz et al., 2017; Leonard et al., 2019; Piai et al., 2018; Scerrati, Mongardi, et al., 2020; Zhang et al., 2013).

### 3.3 Intra‐operative findings

#### 3.3.1 Feasibility and methods

Mapping music was successful in all but one case (93%), in whom music could not be mapped due to occurrence of a stimulation‐induced generalized seizure (Table 2) (Piai et al., 2018). This patient continued to play the violin during surgery without use of cortical stimulation. In the other studies, no surgical complications, related to the intra‐operative music tasks, were reported.

**TABLE 2:** Intra‐operative mapping during music tasks

Authors	Method^a^	Type music task^b^	Additional mapping^c^	Location^d^	Music disruption	Type of disruption	Combined vs. isolated
Bass et al., 2020	Sing	Production and perception	‐	Right pSTG	No	‐	‐
Dziedzic et al., 2020	Keyboard	Production	Motor + Speech/Language	Left posterior MTG + supramarginal gyrus	Yes	Music arrest	Isolated
Garcea et al., 2017	Sing	Production and perception	Speech/Language	(1) Right pSTG (2) Right MTG	(1) Yes (2) No	(1) Music arrest + pitch, rhythm and contour errors (2) No errors	(1) Isolated (2) –
Hegde et al., 2016	Sing	Production, perception and reading	‐	Right STG	No	‐	‐
Katlowitz et al., 2017	Sing	Production	Motor and Speech/Language	(1) Right precentral gyrus (2) Right pSTG	Yes	(1) Music arrest (2) Change melodic contour	(1) Combined with speech/language (2) Isolated
Leonard et al., 2019	Guitar	Production	Speech/Language	(1) Left lateral frontal/parietal (2) Left ventral pre‐central gyrus	(1) No (2) Yes	(1) No errors (2) Music arrest	(1) – (2) Combined with speech/language
Piai et al.,^2018^	Violin^e^	Production	Motor and Speech/Language	Left SMA	No	‐	‐
Roux et al., 2009 (1)	Sing	Production	Motor and Speech/Language	(1) Left precentral gyrus (2) Left Broca region (3) Left opercular ramus (4) Left SMG	(1) Yes (2) No (3) No (4) No	(1) Articulatory (2) No errors (3) No errors (4) No errors	(1) Combined with motor (2) – (3) – (4) –
Roux et al., 2009 (2)	Sing	Production	Speech/Language	(1) Left precentral gyrus (2) Left Broca region (3) Left opercular ramus	(1) Yes (2) No (3) No	(1) Articulatory (2) No errors (3) No errors	(1) Combined with speech/language (2) – (3) –
Roux et al., 2009 (3)	Sing	Production	Motor and Speech/Language	(1) Right precentral gyrus (2) Right MFG	(1) Yes (2) Yes	(1) Articulatory (2) Music arrest	(1) Combined with speech/language (2) Isolated
Roux et al., 2009 (4)	Sing	Production	Speech/Language	(1) Right precentral gyrus (2) Right SMG, MFG	(1) Yes (2) No	(1) Articulatory (2) No errors	(1) Combined with speech/language (2) –
Roux et al., 2009 (5)	Sing	Production	Speech/Language	(1) Right precentral gyrus (2) Right SMG, MFG	(1) Yes (2) No	(1) Loss melodic contour (2) No errors	(1) Combined with speech/language (2) –
Sceratti, Mongardi, et al., 2020	Clarinet	Production	Motor	Right postcentral gyrus	Yes	Music arrest	Combined with motor
Zhang et al., 2013	Sing	Production	Speech/Language	Left MFG/BA 44	Yes	Music arrest	Isolated

Abbreviations: BA, Brodmann's area; MFG, middle frontal gyrus; MTG, medial temporal gyrus; pSTG, posterior superior temporal; SMA, supplementary motor area; SMG, supramarginal gyrus.

^a^ Music tasks during mapping.

^b^ Production: producing music/playing instrument/active singing, Perception: listening to music, Reading: reading music notes.

^c^ Explicit reporting of other non‐music tasks.

^d^ Brain regions mapped during music tasks.

^e^ Mapping not successful due to stimulation‐induced seizure.

Methods of music mapping were vocals (i.e., singing/humming) (*n* = 10, 71%) (Bass et al., 2020; Garcea et al., 2017; Hegde et al., 2016; Katlowitz et al., 2017; Roux et al., 2009; Zhang et al., 2013) or instruments (*n* = 4, 29%) (Dziedzic et al., 2020; Leonard et al., 2019; Piai et al., 2018; Scerrati, Mongardi, et al., 2020). Patients playing instruments included the clarinet (Scerrati, Mongardi, et al., 2020), chords on the guitar (Leonard et al., 2019), simple melodies on the keyboard (Dziedzic et al., 2020) and familiar songs on the violin during surgery (Piai et al., 2018).

In 11 patients (71%), intra‐operative speech/language tasks such as naming and reading were conducted (Dziedzic et al., 2020; Garcea et al., 2017; Katlowitz et al., 2017; Leonard et al., 2019; Roux et al., 2009; Zhang et al., 2013). In six cases, intra‐operative motor function was explicitly reported: one case through finger tapping (Dziedzic et al., 2020), one case with motor and somatosensory‐evoked potentials (MEPS/SSEPS) (Scerrati, Mongardi, et al., 2020) and four merely through observation (Katlowitz et al., 2017; Leonard et al., 2019; Piai et al., 2018; Roux et al., 2009; Scerrati, Mongardi, et al., 2020).

#### 3.3.2 Disruption and localization

**FIGURE 1:**
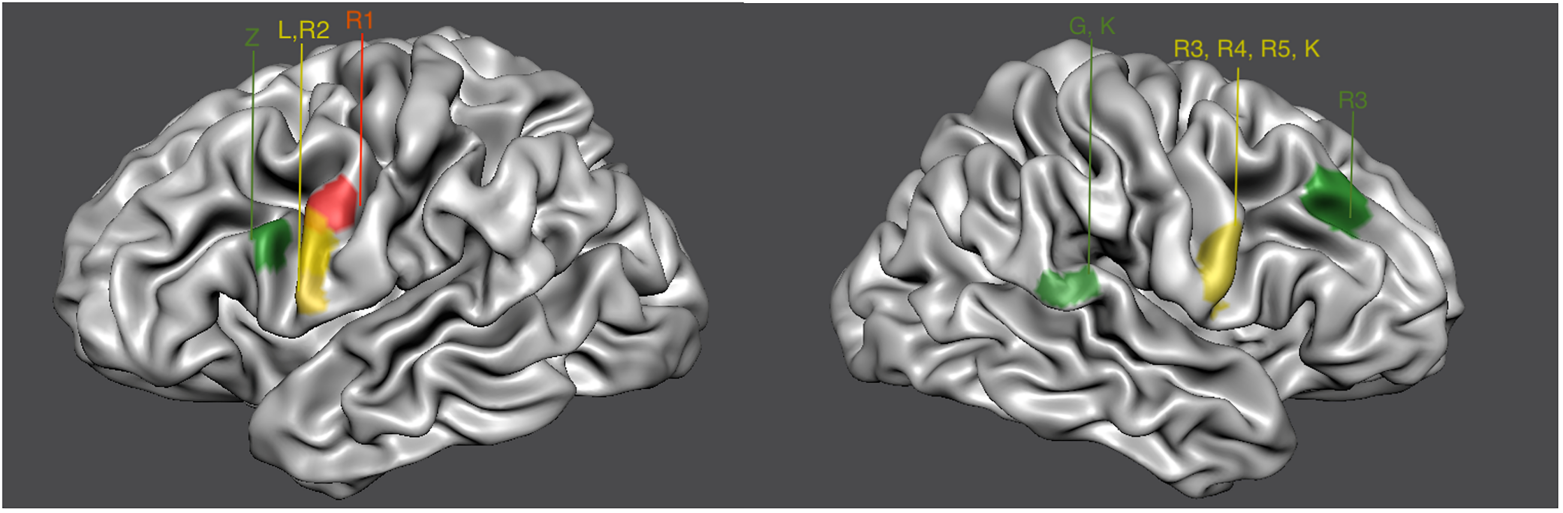
Stimulation sites for music production. Relevant brain regions for music production confirmed by each included case (all right‐handed, except for R1). All methods of music mapping included production except Garcea et al. (G) which included music production and perception. Dziedzic et al. (D) and Scerrati et al. (S) are shown in this figure, due to low specificity of described region and lack of an illustration. Green = brain region with isolated music deficit; confirmed in the right posterior superior temporal gyrus (pSTG) by Garcea et al. (G) and Katlowitz et al. (K), in the right middle frontal gyrus (MFG) by Roux et al. (R3) and in the left MFG (Brodmann's area) by Zhang et al. (Z). Red = brain region with music deficit combined with motor; confirmed by Roux et al. (R1) in the left precentral gyrus. Yellow = brain region with music deficit combined with speech/language, confirmed in the right precentral gyrus by Roux et al. (R3, R4, R5) and Katlowitz et al. (K) and in the left precentral gyrus by Leonard et al. (L) and Roux et al. (R2)

Out of the 13 patients in which music mapping occurred successfully; isolated disruption of musical function was identified in five patients (38%, Table 2) (Dziedzic et al., 2020; Garcea et al., 2017; Katlowitz et al., 2017; Roux et al., 2009; Zhang et al., 2013), only combined with speech/language disruption in four patients (31%) (Leonard et al., 2019; Roux et al., 2009) and with motor disruption in two patients (15%) (Roux et al., 2009; Scerrati, Mongardi, et al., 2020). No music disruption was identified in two patients (15%) (Bass et al., 2020; Hegde et al., 2016). See Figure 1 for all the relevant brain regions with respect to the type of music disruption.

Isolated music disruption occurred in two patients, during singing, when stimulating the right posterior STG with complete music arrest (G) (Garcea et al., 2017) and change in melodic contour (K) (Katlowitz et al., 2017). Isolated music arrest occurred in two patients, during singing, while stimulating the middle frontal gyrus (MFG) in the left (Z) (Zhang et al., 2013) and right (R3) (Roux et al., 2009) hemisphere. Lastly, isolated music disruption occurred during intra‐operative keyboard playing while stimulating the left posterior middle temporal gyrus/supramarginal gyrus (D, not shown in figure as the region was insufficiently specified, with lack of an illustration in the manuscript; Dziedzic et al., 2020).

Music disruption only combined with speech/language occurred in four patients: two patients in the right precentral gyrus during intra‐operative singing, one left‐handed patient with loss of melodic contour combined with affected speech prosody (R5) and one articulatory with naming interference (R4) (Roux et al., 2009), moreover two patients in the left precentral gyrus, one music arrest during intra‐operative guitar playing with repetition errors (L) (Leonard et al., 2019) and one articulatory during intra‐operative singing with naming interference (R2) (Roux et al., 2009).

Music disruption only combined with motor occurred in two patients: one articulatory deficit during intra‐operative singing with motor interference while stimulating the left pre‐central gyrus (R1) (Roux et al., 2009) and one patient while stimulating the right postcentral gyrus with music arrest and dystonic movements in the upper extremities but normal MEPS/SSEPS (S, not shown in figure due to lack of illustration from original manuscript) (Scerrati, Mongardi, et al., 2020).

No music disruption was found in two patients, during intra‐operative singing, while stimulating the right STG (Bass et al., 2020; Hegde et al., 2016).

### 3.4 Peri‐operative course of musicality

#### 3.4.1 Pre‐operative methods

Pre‐operative musical function was assessed in 10 patients (71%) (Bass et al., 2020; Garcea et al., 2017; Hegde et al., 2016; Piai et al., 2018; Roux et al., 2009; Zhang et al., 2013), of which three patients (30%) with use of standardized musical assessment tools such as the Montreal Battery of Evaluation of Amusia (MBEA) (Nunes‐Silva & Haase, 2012), the Seashore Rhythm Test (SRT) (Reitan & Wolfson, 1989) and the Beat Alignment Test (BAT) (Harrison & Mullensiefen, 2018) (Table 3). One study assessed musical function with the MBEA, SRT and BAT (Hegde et al., 2016) while the other two studies report using only the MBEA (Garcea et al., 2017; Zhang et al., 2013). The non‐standardized methods of music assessment (*n* = 7) involved rhythm and tone pitch by the music therapist in one patient (Bass et al., 2020), playing familiar but complex pieces by her own instrument in another patient and one study reported the use of “basic formal testing” in all five patients (R1‐5) (Roux et al., 2009). No pre‐operative deficit in musical function was observed.

**TABLE 3:** Peri‐operative course of musical function

Pre‐operative assessment					
Authors	Assessed	Method	Standardized	Outcome	Localization^a^
Bass et al., 2020	Yes	1. fMRI with passive (A) and active (B) music imagination 2. Neuropsychological rhythm and tonal discrimination 3. Rhythm, tone and pitch test by music therapist	No	1. Voxel activity 2. No deficit 3. No deficit	1. (A) Right STG (B) and left STG 2. – 3. –
Dziedzic et al., 2020	No	‐	‐	‐	‐
Garcea et al., 2017	Yes	1. fMRI with (A) passive piano listening and (B) humming 2. MBEA	Yes	1. Voxel activity 2. 177/180 (98%)	1. (A) Lateral surface right STG and (B) right posterior Sylvian fissure 2. –
Hegde et al., 2016	Yes	1. MBEA 2. SRT 3. BAT 4. Song recognition 5. fMRI while listening familiar (Indian) music	Yes	1. 88–100% 2. 100% 3. 100% 4. No deficit 5. Voxel activity	1. – 2. – 3. – 4. – 5. Bilateral STG
Katlowitz et al., 2017	No	‐	‐	‐	‐
Leonard et al., 2019	No	‐	‐	‐	‐
Piai et al., 2018	Yes	1. Violin/keyboard: major scale and arpeggio 2. Solfege 3. Two easy pieces 4. Reading difficult piece 5. Play familiar but complex piece 6. fMRI: complex motor tasks^b^	No	1. No deficit 2. No deficit 3. No deficit 4. No deficit 5. No deficit 6. No deficit/voxel activity	1. – 2. – 3. – 4. – 5. – 6. Expected precentral/postcentral gyrus
Roux et al., 2009 (1)	Yes	1. “Basic testing”	No	1. NR	1. –
Roux et al., 2009 (2)	Yes	1. “Basic testing”	No	1. NR	1. –
Roux et al., 2009 (3)	Yes	1. “Basic testing”	No	1. NR	1. –
Roux et al., 2009 (4)	Yes	1. “Basic testing”	No	1. NR	1. –
Roux et al., 2009 (5)	Yes	1. “Basic testing”	No	1. NR	1. –
Sceratti, Mongardi, et al., 2020	No	‐	‐	‐	‐
Zhang, 2013	Yes	1. MBEA 2. fMRI (A) humming familiar popular Chinese lyrics and (B) reading notations	Yes	1. 86% 2. Voxel activity	1. – 2. Left MFG and SMA

Abbreviations: BAT, Beat Alignment Test; fMRI, functional magnetic resonance imaging; MBEA, Montreal Battery Evaluation of Amusia; MFG, medial frontal gyrus; SMA, supplementary motor area; SRT, Seashore Rhythm Test; STG, superior temporal gyrus.

^a^ Pre‐operative music localization determination.

^b^ No music tasks were conducted during the fMRI.

Pre‐operative speech/language, evaluated with the use of formal tests in 13 patients (93%) (Bass et al., 2020; Dziedzic et al., 2020; Garcea et al., 2017; Hegde et al., 2016; Katlowitz et al., 2017; Piai et al., 2018; Roux et al., 2009; Scerrati, Mongardi, et al., 2020; Zhang et al., 2013), and motor function, assessed in four patients (29%) (Bass et al., 2020; Piai et al., 2018; Scerrati, Mongardi, et al., 2020; Zhang et al., 2013), revealed no deficits.

Pre‐operative functional MRI (fMRI) for music localization was described in four patients (29%) with music tasks such as listening to music in two patients (Garcea et al., 2017; Hegde et al., 2016), humming familiar songs in one case (Zhang et al., 2013) and passive and active music imagination tasks (i.e., imagining listening or singing) in another case (Bass et al., 2020). Musical dominance (i.e., increased voxel activity) was found in the right STG in one case (Garcea et al., 2017), while bilateral STG activation was found in two other patients during music tasks (Bass et al., 2020; Hegde et al., 2016). Activation of the left MFG and supplementary motor area (SMA) was perceived in the fourth patient during humming, score reading and diverse speech/language tasks (Zhang et al., 2013).

Pre‐operative functional MRI for speech/language localization was described in four patients (Bass et al., 2020; Garcea et al., 2017; Piai et al., 2018), one of which showed less voxel contrast in the right STG compared with the music‐related voxel activity (Garcea et al., 2017), left‐hemispheric dominance in two patients (Bass et al., 2020; Piai et al., 2018) and increased voxel activation in the right anterior temporal lobe during passive word listening tasks (not shown in table) (Bass et al., 2020).

#### 3.4.2 Post‐operative methods

Post‐operative musical function was assessed in seven patients (50%), of which four patients using standardized assessment tools (Table 4): two patients tested with the MBEA (Garcea et al., 2017; Zhang et al., 2013), one with the SRT (Bass et al., 2020) and one with the SRT, MBEA and BAT (Hegde et al., 2016). One patient reported improvement from 86% to 99% on the MBEA attributed to perilesional compensatory activations (Zhang et al., 2013). The other three patients reported similar results compared with baseline, all within normal range (Bass et al., 2020; Garcea et al., 2017). The use of non‐standardized methods for the assessment of musical function after surgery was reported in three patients (23%), in which authors claim that patients were able to play the piano (Dziedzic et al., 2020), the violin (Piai et al., 2018) and the clarinet (Garcea et al., 2017).

**TABLE 4:** Peri‐operative course of musical function

Post‐operative assessment				
Authors	Assessed	Method	Standardized	Outcome
Bass et al., 2020	Yes	1. Pitch recognition 2. Playing guitar 3. SRT	Yes	1. Consistent with baseline 2. “Able to play” 3. Within normal range
Dziedzic et al., 2020	Yes	1. Playing piano	No	“Able to play”
Garcea et al., 2017	Yes	1. Play saxophone upon closure dura 2. MBEA	Yes	1. “Flawless” 2. 175/180 (97%)
Hegde et al., 2016	Yes	1. MBEA 2. SRT 3. BAT 4. Song recognition 5. fMRI in rest	Yes	1. 90–100% 2. 100% 3. 100% 4. “No adverse changes compared with pre‐operative” 5. Enhanced connectivity IFG, MTG, ITG
Katlowitz et al., 2017	No	‐	‐	‐
Leonard et al., 2019	No	‐	‐	‐
Piai et al., 2018	Yes	‐	No	“Patient resumed playing the violin with orchestra, assuming preservation of musical function”
Roux et al., 2009 (1)	No	‐	‐	‐
Roux et al., 2009 (2)	No	‐	‐	‐
Roux et al., 2009 (3)	No	‐	‐	‐
Roux et al., 2009 (4)	No	‐	‐	‐
Roux et al., 2009 (5)	No	‐	‐	‐
Sceratti, Mongardi, et al., 2020	Yes	‐	No	“Resumed playing clarinet in the next 10 months after surgery”
Zhang, 2013	Yes	1. MBEA	Yes	1. 99%

Abbreviations: BAT, Beat Alignment Test; fMRI, functional magnetic resonance imaging; MBEA, Montreal Battery Evaluation of Amusia; MFG, medial frontal gyrus; SMA, supplementary motor area; SRT, Seashore Rhythm Test; STG, superior temporal gyrus.

^a^ Pre‐operative music localization determination.

^b^ No music tasks were conducted during the fMRI.

Post‐operative speech/language was only described in two cases; one patient remained above average on the intelligence and verbal memory tests (Bass et al., 2020). The other patient scored 98% correct, concordant with baseline, on the Aphasia Battery of Chinese test 1 week and 6 months after surgery (Zhang et al., 2013). Furthermore, no reports on other post‐operative neurological deficits were found, except for slight dyscalculia in one case (Piai et al., 2018).

## 4 DISCUSSION

This systematic review supports that mapping music during awake craniotomy is feasible. Moreover, the detection of isolated music disruption in both the right and left hemisphere and preservation of musicality in all patients indicate the additional value of this mapping technique for both hemispheres. Limitations and recommendations for future studies and clinical practice are discussed below.

### 4.1 Feasibility

Almost all included patients (93%) reported successful mapping while performing different music tasks during awake craniotomy. This accounts mostly for singing and humming, as this task was reported in 71% of our included patients and resembles the standard speech/language tasks (Hall et al., 2021; Kanno & Mikuni, 2015). Furthermore, music tasks involved variable instruments, such as the clarinet, keyboard, guitar and violin, all without the occurrence of task‐related complications. While playing these instruments during awake craniotomy therefore seems feasible, generalization of the findings is limited, as different patients may require various positions on the operation table for optimal resection which might interfere with the posture and mobility needed to play for instance the violin. One case failed to map during musical tasks, due to occurrence of a stimulation‐induced seizure (Piai et al., 2018), which was a complication not related to the music task itself. Our results on feasibility should be handled with caution since studies with negative results are often not published and publication bias cannot be ruled out (Montori et al., 2000).

### 4.2 Intra‐operative mapping

Isolated music disruption occurred in 5 out of 14 patients and was identified in the right posterior STG, in both sides the MFG and left middle temporal gyrus suggesting additional value of mapping music in these structures. Isolated music disruption was most often found in the non‐dominant hemisphere (*n* = 3, 60%), but also in the dominant hemisphere (*n* = 2, 40%). This isolated music disruption in the dominant hemisphere is in contrast to the acknowledged hypothesis of Jackson and colleagues explaining that the dominant hemisphere is specialized for speech/language activity and the non‐dominant hemisphere for many non‐linguistic holistic functions such as music perception and production (Bever & Chiarello, 1974; Taylor, 1932). The authors from our included studies that found isolated music disruption in the dominant hemisphere, clarified this with two possible explanations: first, re‐organization to the contra‐lateral side in younger patients combined with loss of function in the non‐dominant hemisphere due to long‐standing lesions (Bass et al., 2020; Hegde et al., 2016). Second, it could be true that both hemispheres are involved in musicality. Indeed, two out of four included patients which performed pre‐operative fMRI found synchronous activation in the right *and* left STG during music imagination tasks, indicating a valuable role for fMRI when operating either side (Bass et al., 2020; Garcea et al., 2017). A fMRI study with healthy participants confirmed this, showing increased voxel activation during music listening in both the right *and* left STG (Angulo‐Perkins et al., 2014). Furthermore, our included studies suggest additional value of pre‐operative fMRI as music localization was confirmed in three out of these four cases (Garcea et al., 2017; Hegde et al., 2016; Zhang et al., 2013).

Speech/language and music errors were found in 4 out of 14 patients when stimulating both the left and right precentral gyrus, suggesting a speech/language‐induced musical disruption. We observed this speech/language‐induced musical disruption more during intra‐operative singing (*n* = 3, 75%) as opposed to playing an instrument (*n* = 1, 25%). This might be explained by the several common characteristics of speech/language and singing, such as their hierarchical structure and prosodic features (e.g., phrase‐final lengthening) (Heffner & Slevc, 2015; Patel, 2003). However, the small numbers limit firm conclusions on this relation. In two patients, music disruption was found combined with visible motor contractions in the right post‐central gyrus and left pre‐central gyrus. These regions can therefore not solely be devoted to the function of music (DiGuiseppi & Tadi, 2021; Scerrati, Mongardi, et al., 2020). Eight cases did not explicitly mention their findings on motor mapping, so we assumed no motor deficits in these patient, as motor disruption can be determined through mere observation. Future studies should carefully describe each task per brain region, to enable readers to understand the origin (motor, speech/language or merely music) of the deficit.

### 4.3 Preservation of musical function

All the included cases in this study demonstrated preserved musical function, indicative for added value of intra‐operative music tasks during awake craniotomy. We do acknowledge that, in the literature, we did not find any case reports describing amusia after awake craniotomy without music tasks. However, literature describes post‐operative amusia in one case after resection of the right‐sided gyrus of Heschl (Russell & Golfinos, 2003) and a non‐aphasic singer which lost his capacity to sing after resection of a cyst in the right MFG (Mann, 1898). These studies, which confirm our cases which found isolated music disruption in the right MFG and STG, convince us of the added value of testing musicality during awake craniotomy. Our data on the postoperative follow‐up were limited to only 7 out of 14 cases. Furthermore, while these studies reported patients playing their instrument after surgery, objective standardized tools were only used in four studies challenging comparisons between preoperative and postoperative musicality (Nunes‐Silva & Haase, 2012). Future studies should therefore (a) report follow‐up data and (b) use an objective, standardized assessment tool.

### 4.4 Strengths and limitations

This study has several strengths and limitations. This is the first systematic review to assess the feasibility and value of music mapping during awake craniotomy. We schematically presented an overview of all the different methods, brain regions of interest and peri‐operative course. Our findings are intended to be used as a guidance for clinical practice and for future studies. Our conclusions with respect to feasibility should be handled with caution, as all studies had positive outcomes (successful music mapping with preserved postoperative function) possibly indicating publication bias. Moreover, our small sample size, lack of control group, different methods of assessing musical function, and limited information concerning post‐operative musical function make it difficult to draw firm conclusions on the true additional value of mapping music for preserving musicality.

### 4.5 Recommendations

Publication of unsuccessful case reports should be encouraged to improve insights in the feasibility of musical performance during awake craniotomy. Second, although intra‐operative music tasks may vary, disruption of music should always be compared with speech/language and/or motor tasks to understand the origin (motor, speech/language or merely music) of the deficit. Third, musicality should also be assessed with a standardized objective scoring form before and after surgery allowing comparison between several moments and studies. Lastly, pre‐operative fMRI with musicality related tasks is desirable in order to improve knowledge on the localization of music in neurosurgical patients and to allow for better interpretation of the intra‐operative findings.

### 4.6 Conclusions

Successful mapping during music tasks in all but one reported patient shows the feasibility of intra‐operative mapping of musical function. Moreover, isolated music disruption in both the right and left hemisphere with preservation of musicality in all patients indicate an added value of this mapping technique for both hemispheres. Future studies should use standardized protocols as described above to assess the true feasibility and added value of mapping music during awake craniotomy.

## CONFLICTS OF INTEREST STATEMENT

Nothing to disclose.

## AUTHOR CONTRIBUTIONS

This study was funded by the Erasmus University Medical Center. The funders of the study had no role in the study design, data collection, data analysis, manuscript preparation and publication decision. PK coordinated this study. PK and TB conducted the literature search, extracted the data, conducted the statistical analysis and wrote the first draft of the manuscript. PK, AV, DS, CD, JJ and MK interpreted the data and critically revised the manuscript. PK, TB, AV, DS, CD, JJ and MK had full access to all of the data in the study and can take responsibility for the integrity of the data and the accuracy of the data analysis.

### PEER REVIEW

The peer review history for this article is available at https://publons.com/publon/10.1111/ejn.15559.

## Data Availability

Data sharing is not applicable to this article as no new data were created in this study. The authors confirm that the data supporting the findings of this study are available within the article and its supplementary materials.

## APPENDIX A.

### SEARCH AWAKE SURGERY MUSICIANS (DATE 26 MARCH 2021)

**Table:**

Database searched	Via	Years of coverage	Records	Records after duplicates removed
Embase	Embase.com	1971–present	525	516
Medline ALL	Ovid	1946–present	276	35
Web of Science Core Collection^a^	Web of knowledge	1975–present	308	88
Cochrane Central Register of Controlled Trials	Wiley	1992–present	55	21
**Total**			**1164**	**660**

^a^Science Citation Index Expanded (1975–present); Social Sciences Citation Index (1975–present); Arts & Humanities Citation Index (1975–present); Conference Proceedings Citation Index‐Science (1990–present); Conference Proceedings Citation Index‐Social Science & Humanities (1990–present); Emerging Sources Citation Index (2015–present).

**Embase.com**

(‘awake surgery’/de OR ‘awake craniotomy’/de OR (craniotomy/de AND wakefulness/de) OR (‘brain surgery’/exp AND ‘brain mapping’/de) OR ‘operating room’/de OR ‘central nervous system tumor’/exp/dm_su OR ‘arteriovenous malformation’/exp/dm_su OR epilepsy/exp/dm_su OR ‘auditory brain stem implantation’/exp OR ‘nerve surgery’/exp OR ‘neuroendoscopy’/exp OR ‘neuronavigation’/exp OR ‘skull surgery’/exp OR ‘ventriculostomy’/exp OR ‘glioma’/exp/dm_su OR ‘brain depth stimulation’/de OR (((awake) NEAR/3 (surger* OR surgical*)) OR neurosurger* OR Trephin* OR craniotom* OR cranioplast* OR craniectom* OR lobectom* OR trepanat* OR ((brain* OR cerebr* OR cerebell* OR nerve* OR cranial* OR skull* OR hydrocephal* OR meningi* OR gangliogliom* OR schwannom* OR astrocytom* OR Acoustic‐neurom* OR Chordom* OR Lymphom* OR Craniopharyngiom* OR Ependymom* OR Medulloblastom* OR epilep* OR posterior‐fossa* OR subdural* OR epidural* OR subarachnoidal* OR intraparenchym* OR intra‐parenchym* OR ventricul* OR neurooncol* OR Intracranial OR cortical* OR cortex* OR cerebral OR neuro* OR lobe OR lobar OR glio* OR avm OR arteriovenous‐malformati* OR cns OR central‐nervous‐system) NEAR/3 (surger* OR resection* OR operative* OR intraoperative* OR resection* OR operative* OR intraoperative*)) OR (operati* NEAR/3 (room* OR theat*)) OR ventriculostom* OR lobectom* OR (brain NEAR/3 (depth OR deep) NEAR/3 stimulat*) OR craniosynostos*):ab,ti,kw) AND (musician/exp OR singer/de OR (musician* OR pianist* OR violin* OR piano* OR guitar* OR koto OR shamisen OR flute OR saxophone OR singer* OR singing* OR clarinet* OR ((music* OR vocal) NEAR/3 (perform* OR playing OR artist* OR mapping OR skill* OR instrument* OR function* OR production* OR region*)) OR ((wind OR string) NEAR/3 (instrument*)) OR musicalit* OR musicolog*):ab,ti,kw)

**Medline ALL Ovid**

((Craniotomy /AND Wakefulness/) OR (Brain Mapping/AND Surgical Procedures, Operative/) OR Operating Rooms/ OR exp Central Nervous System Neoplasms/su OR exp Arteriovenous Malformations/su OR exp Epilepsy/su OR Auditory Brain Stem Implantation/ OR nerve surgery/ OR Neuroendoscopy/ OR Neuronavigation/ OR Ventriculostomy/ OR (((awake) ADJ3 (surger* OR surgical*)) OR neurosurger* OR Trephin* OR craniotom* OR cranioplast* OR craniectom* OR lobectom* OR trepanat* OR ((brain* OR cerebr* OR cerebell* OR nerve* OR cranial* OR skull* OR hydrocephal* OR meningi* OR gangliogliom* OR schwannom* OR astrocytom* OR Acoustic‐neurom* OR Chordom* OR Lymphom* OR Craniopharyngiom* OR Ependymom* OR Medulloblastom* OR epilep* OR posterior‐fossa* OR subdural* OR epidural* OR subarachnoidal* OR intraparenchym* OR intra‐parenchym* OR ventricul* OR neurooncol* OR Intracranial OR cortical* OR cortex* OR cerebral OR neuro* OR lobe OR lobar OR glio* OR avm OR arteriovenous‐malformati* OR cns OR central‐nervous‐system) ADJ3 (surger* OR resection* OR operative* OR intraoperative* OR resection* OR operative* OR intraoperative*)) OR (operati* ADJ3 (room* OR theat*)) OR ventriculostom* OR lobectom* OR (brain ADJ3 (depth OR deep) ADJ3 stimulat*) OR craniosynostos*).ab,ti,kf.) AND (musician/OR singer/OR (musician* OR pianist* OR violin* OR piano* OR guitar* OR koto OR shamisen OR flute OR saxophone OR singer* OR singing* OR clarinet* OR ((music* OR vocal) ADJ3 (perform* OR playing OR artist* OR mapping OR skill* OR instrument* OR function* OR production* OR region*)) OR ((wind OR string) ADJ3 (instrument*)) OR musicalit* OR musicolog*).ab,ti,kf.)

**Web of Science (Social Sciences Citation Index/Science Citation Index Expanded)**

TS=(((((awake) NEAR/2 (surger* OR surgical*)) OR neurosurger* OR Trephin* OR craniotom* OR cranioplast* OR craniectom* OR lobectom* OR trepanat* OR ((brain* OR cerebr* OR cerebell* OR nerve* OR cranial* OR skull* OR hydrocephal* OR meningi* OR gangliogliom* OR schwannom* OR astrocytom* OR Acoustic‐neurom* OR Chordom* OR Lymphom* OR Craniopharyngiom* OR Ependymom* OR Medulloblastom* OR epilep* OR posterior‐fossa* OR subdural* OR epidural* OR subarachnoidal* OR intraparenchym* OR intra‐parenchym* OR ventricul* OR neurooncol* OR Intracranial OR cortical* OR cortex* OR cerebral OR neuro* OR lobe OR lobar OR glio* OR avm OR arteriovenous‐malformati* OR cns OR central‐nervous‐system) NEAR/2 (surger* OR resection* OR operative* OR intraoperative* OR resection* OR operative* OR intraoperative*)) OR (operati* NEAR/2 (room* OR theat*)) OR ventriculostom* OR lobectom* OR (brain NEAR/2 (depth OR deep) NEAR/2 stimulat*) OR craniosynostos*)) AND ((musician* OR pianist* OR violin* OR piano* OR guitar* OR koto OR shamisen OR flute OR saxophone OR singer* OR singing* OR clarinet* OR ((music* OR vocal) NEAR/2 (perform* OR playing OR artist* OR mapping OR skill* OR instrument* OR function* OR production* OR region*)) OR ((wind OR string) NEAR/2 (instrument*)) OR musicalit* OR musicolog*)))

**Cochrane CENTRAL register of trials**

((((awake) NEAR/3 (surger* OR surgical*)) OR neurosurger* OR Trephin* OR craniotom* OR cranioplast* OR craniectom* OR lobectom* OR trepanat* OR ((brain* OR cerebr* OR cerebell* OR nerve* OR cranial* OR skull* OR hydrocephal* OR meningi* OR gangliogliom* OR schwannom* OR astrocytom* OR Acoustic‐neurom* OR Chordom* OR Lymphom* OR Craniopharyngiom* OR Ependymom* OR Medulloblastom* OR epilep* OR posterior‐fossa* OR subdural* OR epidural* OR subarachnoidal* OR intraparenchym* OR intra‐parenchym* OR ventricul* OR neurooncol* OR Intracranial OR cortical* OR cortex* OR cerebral OR neuro* OR lobe OR lobar OR glio* OR avm OR arteriovenous‐malformati* OR cns OR central‐nervous‐system) NEAR/3 (surger* OR resection* OR operative* OR intraoperative* OR resection* OR operative* OR intraoperative*)) OR (operati* NEAR/3 (room* OR theat*)) OR ventriculostom* OR lobectom* OR (brain NEAR/3 (depth OR deep) NEAR/3 stimulat*) OR craniosynostos*):ab,ti,kw) AND ((musician* OR pianist* OR violin* OR piano* OR guitar* OR koto OR shamisen OR flute OR saxophone OR singer* OR singing* OR clarinet* OR ((music* OR vocal) NEAR/3 (perform* OR playing OR artist* OR mapping OR skill* OR instrument* OR function* OR production* OR region*)) OR ((wind OR string) NEAR/3 (instrument*)) OR musicalit* OR musicolog*):ab,ti,kw)
